# Supplementary material for: Predictions on the Phase Constitution of SmCo7−XMx Alloys by Data Mining
Source: Nanomaterials (Basel). 2022 Apr 24;12(9):1452. doi: 10.3390/nano12091452 (PMC9100234; doi:10.3390/nano12091452)
Supplement: Supplementary file 1 [file nanomaterials-12-01452-s001.zip › nanomaterials-1654402 - Supplementary Materials.pdf]

nanomaterials

MDPI

Supplementary Materials

Predictions on the Phase Constitution of $SmCo_{7-x}M_x$ Alloys by Data Mining

Guojing Xu, Hao Lu *, Kai Guo, Fawei Tang and Xiaoyan Song *

Faculty of Materials and Manufacturing, Key Laboratory of Advanced Functional Materials, Ministry of Education of China, Beijing University of Technology, Beijing 100124, China; GJ.xu@emails.bjut.edu.cn (G.X.); kevin_gk@emails.bjut.edu.cn (K.G.); faweitang@bjut.edu.cn (F.T.)
* Correspondence: haolu@bjut.edu.cn (H.L.); xysong@bjut.edu.cn (X.S.)

Table S1. The symbols and meanings involved in the article.

Symbol	Meaning
M	doping elements
$\boldsymbol{x_c}$	the percentage of doping element substituting Co ($\boldsymbol{x_c = 100x/7}$)
$\boldsymbol{x_{sub}}$	the proportion of doping element substituting Co ($\boldsymbol{x_{sub} = x/7}$)
$\boldsymbol{X_{form}}$	material form
$\boldsymbol{X_{proc}}$	material preparation processes
$\boldsymbol{X^*_{proc}}$	material preparation processes after further divide
d	the average grain size of material
lgd	logarithm of the grain size
$\boldsymbol{X_M}$	the intrinsic properties of elements
C, σ	hyperparameters of SVM model

Table S2. The optimal features and the mean AUC with different number of features.

Number of features	Features	Mean AUC
0	BF	0.65
1	BF, $x_{sub} \cdot \mathrm{T_m\_Co}$	0.75
**2**	**BF, $\boldsymbol{x_{sub} \cdot \mathrm{T_m}}$, $\boldsymbol{x_{sub} \cdot \chi_{\mathrm{Co}}}$**	**0.78**
3	BF, $x_{sub} \cdot \mathrm{T_m\_Co}$, $x_{sub} \cdot r_a\mathrm{\_Co}$, $x_{sub} \cdot \kappa$	0.79
4	BF, $x_{sub} \cdot A_r$, $x_{sub} \cdot \mathrm{T_m\_Co}$, $x_{sub} \cdot \kappa$, $x_{sub} \cdot \chi\mathrm{\_Sm}$	0.80
5	BF, $x_{sub} \cdot A_r$, $x_{sub} \cdot \mathrm{E_{i,1st}\_Sm}$, $x_{sub} \cdot \mathrm{T_m\_Sm}$, $x_{sub} \cdot r_a\mathrm{\_Sm}$, $x_{sub} \cdot \chi$	0.80
6	BF, $x_{sub} \cdot A_r$, $x_{sub} \cdot \mathrm{E_{i,1st}\_Sm}$, $x_{sub} \cdot \mathrm{T_m\_Sm}$, $x_{sub} \cdot r_a$, $x_{sub} \cdot \chi$, $x_{sub} \cdot \Delta H_f$	0.79
7	BF, $x_{sub} \cdot A_r$, $x_{sub} \cdot \mathrm{E_{i,1st}\_Sm}$, $x_{sub} \cdot \mathrm{T_m\_Sm}$, $x_{sub} \cdot r_a$, $x_{sub} \cdot \chi\mathrm{\_Sm}$, $x_{sub} \cdot \Delta H_f$, $x_{sub} \cdot \kappa\_Co$	0.78

To simplify the writing, $\mathrm{X_M\_Co}$ denotes $|X_{Co} - X_M|$, and $\mathrm{X_M\_Sm}$ denotes $|X_{Sm} - X_M|$

Table S3. Design of independent variable space.

	$\boldsymbol{M}$	$\boldsymbol{x_C}$	$\boldsymbol{lgd}$ / nm	$\boldsymbol{C^*_{proc}}$	$\boldsymbol{C_{form}}$
Range	B, Al, C…Fe	0–17.5%	1–3	Preparation, Heat treatment	Ribbon, Bulk, Powder
Step	-	0.05%	0.1	-	-
Count	35	350	21	2	3

Table S4. LTECV grouping statistics.

Group	Hf/Fe	Zr/Al	Ga/C	Ti/Ta	Ag/V	Si/Mn	Nb/Cr	Mo/Sn	Ge/B	Cu/Ni
All	35	35	35	29	27	31	28	29	23	27
Other	31	24	23	21	22	18	19	15	20	16
Sum	66	59	58	50	49	49	47	44	43	43
